# Wavelength-Tunable and Water-Stable Cesium–Lead-Based All-Bromide Nanocrystal–Polymer Composite Films Using Ultraviolet-Curable Prepolymer as an Anti-Solvent

**DOI:** 10.3390/polym14030381

**Published:** 2022-01-19

**Authors:** Wook Hyun Kim, Jungyoun Bae, Kang-Pil Kim, Sungho Woo

**Affiliations:** 1 Division of Energy Technology, Daegu Gyeongbuk Institute of Science and Technology (DGIST), 333 Techno Jungang-daero, Dalseong-gun, Daegu 42988, Korea; kwh1980@dgist.ac.kr (W.H.K.); bjy9606@knu.ac.kr (J.B.); kkp@dgist.ac.kr (K.-P.K.); 2 Department of Chemical Engineering, Kyungpook National University, 80 Daehak-ro, Buk-gu, Daegu 41566, Korea

**Keywords:** cesium halide perovskite nanocrystals, blue emission, CsPbBr_3_/Cs_4_PbBr_6_, UV-curable prepolymer, anti-solvent, reprecipitation

## Abstract

All-inorganic metal halide perovskite nanocrystals (IPeNCs) have become one of the most promising luminescent materials for next-generation display and lighting technology owing to their excellent color expression ability. However, research on IPeNCs with stable blue emission is limited. In this paper, we report stable blue emissive all-bromide IPeNCs obtained through a modified ligand-assisted reprecipitation method using an ultraviolet (UV)-curable prepolymer as the anti-solvent at a low temperature. We found that the blue emission originates from quantum-confined CsPbBr_3_ nanoparticles formed together with the colorless wide-bandgap Cs_4_PbBr_6_ nanocrystals. When the temperature of the prepolymer was increased from 0 to 50 °C, CsPbBr_3_ nanoparticles became larger and more crystalline, thereby altering their emission color from blue to green. The synthesized all-bromide blue-emitting IPeNC solution remained stable for over 1 h. It also remained stable when it was mixed with the green-emitting IPeNC solution. By simply exposing the as-synthesized IPeNC–prepolymer solutions to UV light, we formed water-stable composite films that emitted red, green, blue, and white colors. We believe that this synthetic method can be used to develop color-emitting composite materials that are highly suitable for application as the color conversion films of full-color liquid crystal display backlight systems and lighting applications.

## 1. Introduction

Recently, all-inorganic metal halide perovskite nanocrystals (IPeNCs) with a general formula of CsPbX_3_ (X = Cl^−^, Br^−^, I^−^) have attracted intense interest for next-generation light-emitting diodes (LEDs) and full-color displays owing to their outstanding optical properties, such as a tunable bandgap, high photoluminescence (PL) quantum yield, and narrow PL linewidths [1,2]. Ligand-assisted reprecipitation (LARP) and hot injection (HI) methods have been widely used to synthesize IPeNCs. The HI method has several obvious drawbacks for commercialization, such as the need for a high temperature, vacuum, and inert atmosphere. In contrast, LARP is a simpler approach that can be conducted under an air atmosphere at relatively low temperatures; thus, it is more convenient for large-scale production [3].

In LED and display applications, the three primary colors of red, green, and blue (RGB) are essential for the realization of full and wide color gamuts. However, the development of IPeNCs with bright, stable blue emission remains a key problem. To address this, four main strategies have been pursued to develop blue-emitting IPeNCs [4,5]. The most widely used approach is the use of a mixed Br/Cl halide composition to obtain CsPbBr_x_Cl_3–x_ IPeNCs [2]. However, when these Br/Cl mixed halide IPeNCs are exposed to light or an electrical potential, they undergo significant phase separation, which results in unavoidable color instability and decreased emission intensity. This phase separation is induced by their lattice mismatch, which is due to the difference in the atomic radii of Cl and Br. The second method is doping small metal cations (Mn^2+^, Sn^2+^, Cd^2+^, Zn^2+^, Cu^2+^, Al^3+^, Nd^3+^, Bi^3+^, Sb^3+^) into Pb sites to distort the lattice or tilt the PbBr_6_ octahedra (or both), thereby shifting the PL emission to the blue region [6,7,8,9,10,11]. However, some dopants generate dual emissions, such as a blue region with an extra region from the doped cations. Another strategy is to impose a strong quantum confinement effect by reducing the radius of CsPbBr_3_ IPeNCs to less than the exciton Bohr radius (approximately 7 nm for CsPbBr_3_). The fourth method is the synthesis of two-dimensional (2D) CsPbBr_3_ nanoplatelets that exhibit a quantum confinement effect. However, consistent fabrication is challenging for both quantum-confined nanocrystals (NCs) and 2D shapes owing to the phase transition from low-dimensional nanostructures to high-dimensional perovskites [12,13,14,15,16].

IPeNCs are unstable in water or polar solvents owing to their intrinsic ionic bonding, which is another key obstacle to their use in optoelectronic device applications. To overcome this issue, several studies have attempted to encapsulate IPeNCs using inorganic silica, TiO_2_, a mesoporous alumina structure, organic ligands, and polymer matrix materials [17,18,19,20]. Recently, IPeNCs have been composited with polymers to enhance their stability against moisture, light, and heat. Compared with other techniques, blending IPeNCs with a commercial polymer, such as polymethylmethacrylate, poly(laurylmethacrylate), polydimethylsiloxane, polystyrene, poly(styrene–ethylene–butylene–styrene), and thermoplastic polyurethane, is a relatively easy and effective method for protecting IPeNCs [21,22,23,24,25,26,27]. However, during the time-consuming blending process in air, IPeNCs often decompose and agglomerate owing to the large difference in the polarity between the IPeNCs and polymers. Hence, in situ synthesis strategies using ultraviolet (UV)-curable prepolymers as solvents or ligands to prevent the IPeNCs from agglomerating within the polymer have been extensively studied [28,29,30,31,32].

In this study, for the first time, we develop emission-wavelength-tunable all-bromide-based IPeNC–polymer composite films obtained through a modified LARP synthesis method using a UV-curable prepolymer as the anti-solvent. The absorption spectral and X-ray diffraction (XRD) data showed that the nanoparticles synthesized using the prepolymer comprised Cs_4_PbBr_6_ and CsPbBr_3_ NCs. In addition, a blue luminescent solution of these all-bromide NCs synthesized from the prepolymer was stable for more than 1 h. Moreover, after being mixed with a green luminescent CsPbBr_3_ solution, the mixture exhibited stable blue and green luminescence peaks, which remained discrete and did not merge for more than 1 h. After UV curing, the IPeNC–polymer composite films not only provided an adjustable emission color from blue (465 nm) to green (515 nm) without the introduction of mixed anion composition but also exhibited high stability in water. Therefore, we believe that our all-bromide-based IPeNC–polymer composite films have high potential for the color conversion film of liquid crystal display (LCD) backlight systems and lighting applications.

## 2. Materials and Methods

### 2.1. Materials

Lead bromide (PbBr_2_, 99.999%), cesium bromide (CsBr, 99.999%), oleic acid (OA, technical grade, 90%), dimethylformamide (DMF, anhydrous, 99.8%), toluene (anhydrous, 99.8%), benzyl methacrylate (BMA, 96%), trimethylolpropane triacrylate (TMPTA), and a blend (50/50 wt.%) of diphenyl(2,4,6-trimethylbenzoyl)phosphine oxide (TPO) and 2-hydroxy-2-methylpropiophenone (HMPP) were purchased from Sigma-Aldrich (Saint Louis, MO, USA). Oleylamine (OAm, 95%) was purchased from Strem (Newburyport, MA, USA). All chemicals were used as-received without further purification. The UV LED (395 nm, 10 W) was purchased from JIN LED Co. (Seoul, Korea).

### 2.2. Preparation of the Precursors and Prepolymer

The metal halide precursor solution was prepared by dissolving 0.2 mmol of CsBr and 0.2 mmol of PbBr_2_ in 5 mL of DMF solvent under vigorous stirring at 90 °C. Subsequently, 0.375 mL of OA and 0.25 mL of OAm were added to stabilize the precursor solution. Then, the solution was stirred at 90 °C to complete the dissolution prior to injecting it into the anti-solvent. The UV-curable prepolymer was prepared by mixing BMA (monomer), TMPTA (crosslinker), and TPO/HMPP (photoinitiator) in volume fractions of 98:1:1.

### 2.3. Synthesis of IPeNCs and Its Composite Films

The IPeNCs were synthesized by the simple one-step LARP method first reported by Li et al. [3] with some modifications. Prior to injection, the precursor solution was heated to 90 °C to attain homogeneity, and the temperature of the anti-solvent (toluene or prepolymer) was maintained at 0, 25, or 50 °C using a temperature-controllable stirrer plate. Subsequently, 0.2 mL of the precursor solution at 90 °C was separately injected into 3 mL of the anti-solvent at 0, 25, or 50 °C, under vigorous stirring for 10 min (Figure S1). Hereinafter, these synthesized solutions are labeled by the anti-solvent with a suffix indicating their temperature, namely, prepolymer-0, prepolymer-25, prepolymer-50, toluene-0, and toluene-25.

The prepared IPeNC–prepolymer solutions were each coated on a glass substrate and exposed to 365 nm UV light (10 mW) for 10 min to crosslink the BMA monomer, thereby forming an IPeNC–polymer composite film.

### 2.4. Characterization and LED Color Measurements

The UV-visible (UV-vis) absorption spectra were measured using a UV-3600 spectrophotometer (Shimadzu Co., Kyoto, Japan). The PL spectra were collected using an RF-6000 spectrofluorophotometer (Shimadzu Co., Kyoto, Japan) at an excitation wavelength of 350 nm. The PL quantum yields (PLQYs) of the samples were measured using an integrated sphere (FluoroMax-4, Horiba Scientific, Kyoto, Japan) at an excitation wavelength of 365 nm. XRD patterns were recorded using an X’pert PRO diffractometer (PANalytical, Malvern, UK) with Cu Kα radiation (wavelength = 1.540 Å) to determine the crystal structure of the nanoparticles. A Rietveld refinement was performed to quantitatively analyze these patterns using the Highscore Plus software suite (PANalytical, Malvern, UK). Transmission electron microscopy (TEM) images were obtained using an FEI Technai G2 F20 TEM system (FEI, Hillsboro, OR, USA). A laboratory-made color measurement system equipped with a PR-670 spectrophotometer (Photo Research Co., North Syracuse, NY, USA) was used to measure the color coordinates and temperature. The dipole moment and molecular structure of BMA, toluene, and other chemicals were calculated by PM6 semi-empirical methods using the MOPAC2016 software [33,34]. The dipole moments are given in Debye units (1 D = 3.334 × 10^−30^ C·m).

## 3. Results and Discussion

**Figure 1:**
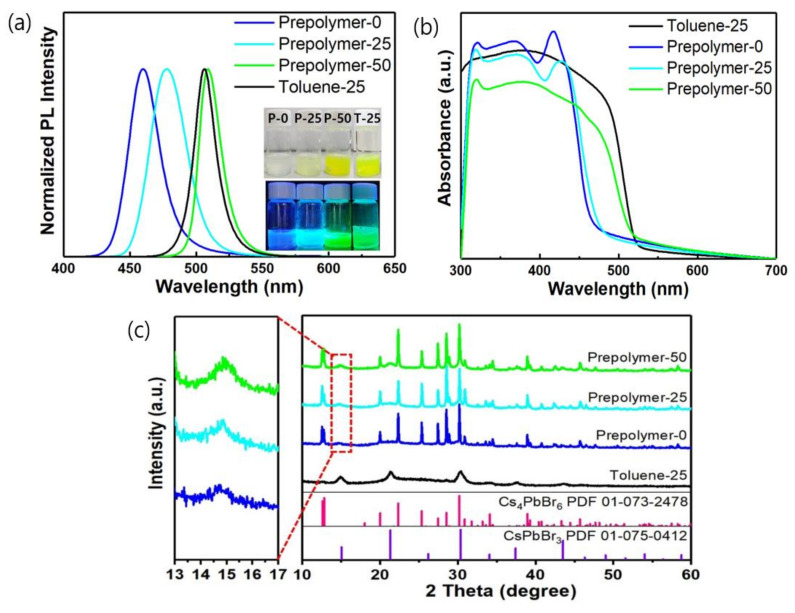
(**a**) Normalized photoluminescence (PL) spectra, (**b**) absorption spectra, and (**c**) X-ray diffraction (XRD) patterns of prepolymer-0, prepolymer-25, prepolymer-50, and toluene-25. The insets in (**a**) show images of the as-synthesized samples under ambient light (upper) and ultraviolet (UV) light (lower).

In the LARP method, the selection of suitable anti-solvents and injection temperature are two of the main factors that determine the crystal structure, size, and shape of the final IPeNCs. As shown in Figure 1a, the PL spectrum shifted from green (513 nm) to blue (465 nm) as the prepolymer (anti-solvent) temperature decreased. The measured PLQY values of prepolymer-0, prepolymer-25, prepolymer-50, and toluene-25 are 29%, 57%, 38%, and 40%, respectively. These results indicate that blue-emitting (465 nm) IPeNCs with a PLQY of 29% were successfully synthesized by our modified LARP method using the prepolymer as the anti-solvent.

**Figure 2:**
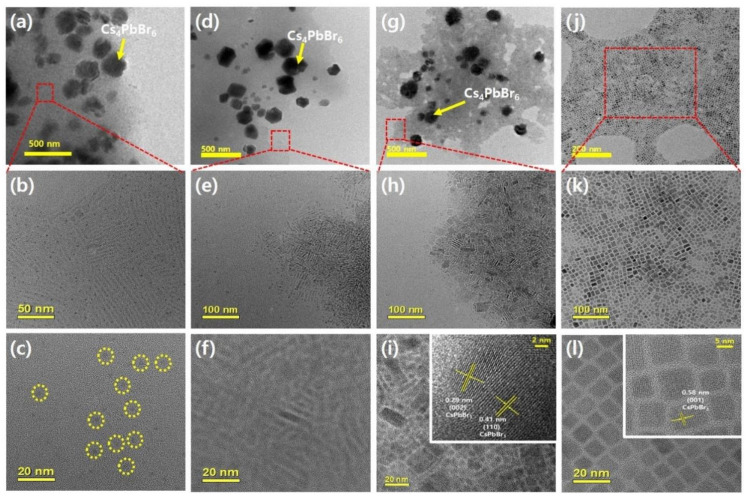
Transmission electron microscopy (TEM) images of the all-inorganic metal halide perovskite nanocrystals (IPeNCs) in (**a**–**c**) prepolymer-0, (**d**–**f**) prepolymer-25, (**g**–**i**) prepolymer-50, and (**j**–**l**) toluene-25. (**a**,**d**,**g**,**j**) are low-magnification images, and (**b**,**c**,**e**,**f**,**h**,**i**,**k**,**l**) are enlarged images of the CsPbBr_3_ nanoparticles. The yellow dotted circles in (**c**) indicate small amorphous CsPbBr_3_ nanoparticles. The insets in (**i**,**j**) show the lattice constants of CsPbBr_3_ nanoparticles.

In Figure 1b, the distinct absorption peak at 314 nm indicates the formation of Cs_4_PbBr_6_ NCs when the metal halide precursor was injected into the prepolymer solution at 0 °C [35], whereas the excitonic absorption peak near 430 nm indicates the formation of quantum-confined CsPbBr_3_ NCs. In the case of prepolymer-25, the absorption peak at 430 nm shifted to a higher wavelength, and the absorption edge red-shifted. In prepolymer-50, the excitonic absorption peak disappeared, and the CsPbBr_3_ absorption edge shifted significantly to 515 nm. Moreover, in toluene-25, the absorption peak of Cs_4_PbBr_6_ and the excitonic absorption peak of CsPbBr_3_ were not observed, and only the absorption edge of CsPbBr_3_ at 515 nm was detected, which indicates that only the CsPbBr_3_ phase formed in the toluene system. This result is consistent with the trends in the XRD peaks shown in Figure 1c and the TEM images in Figure 2j–l. Specifically, the specific XRD peaks of Cs_4_PbBr_6_ and CsPbBr_3_ simultaneously appeared in the prepolymer system, whereas toluene-25 shows only the CsPbBr_3_ diffraction pattern [35,36,37]. Moreover, the XRD peak of the Cs_4_PbBr_6_ phase was more intense than that of the CsPbBr_3_ phase in the prepolymer system, indicating that Cs_4_PbBr_6_ crystals were larger than the CsPbBr_3_ crystals. In addition, the weak, broad XRD peak of CsPbBr_3_ at approximately 15° in the prepolymer-0 sample indicates that the CsPbBr_3_ phase was nearly amorphous with extremely small grains, as confirmed by the TEM images in Figure 2c [3]. Thus, the IPeNCs in prepolymer-0 were approximately 4 nm in size and existed in a nearly amorphous phase. In addition, the CsPbBr_3_ diffraction peak intensified with the increase in the prepolymer temperature, implying that the CsPbBr_3_ nanoparticles grew larger with the increasing prepolymer temperature, as shown in Figure 2a–i. Figure S3 shows the particle size distribution of the CsPbBr_3_ nanoparticles measured from the TEM image in Figure 2. The average particle sizes of the CsPbBr_3_ nanoparticles in prepolymer-0, prepolymer-25, prepolymer-50, and toluene-25 were 3.4, 6.2, 10.2, and 10.1 nm, respectively. A Rietveld refinement was applied to the XRD data shown in Figure 1c to calculate the compositional ratio of Cs_4_PbBr_6_ to CsPbBr_3_ in each sample (see Figure S4 and Table S1), which confirmed the coexistence of Cs_4_PbBr_6_ and CsPbBr_3_ as the major and minor components in the prepolymer system, respectively. In contrast, toluene-25 consisted of only CsPbBr_3_ nanoparticles. In addition, the proportion of CsPbBr_3_ slightly increased as the setting temperature of the prepolymer increased, which is consistent with the increase in the crystallinity of CsPbBr_3_ nanoparticles with the increasing setting temperature. Moreover, Cs_4_PbBr_6_ is known to exhibit no obvious absorption or emission in the visible light region, because it has a wide bandgap of 3.9 eV [35,36,37].

**Figure 3:**
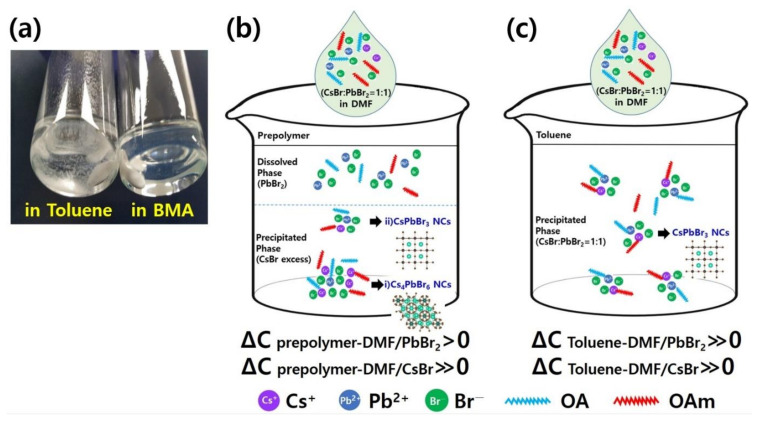
(**a**) Solubility test with 0.2 mL of the PbBr_2_–dimethylformamide (DMF) solution (0.2 mmol of PbBr_2_ in 5 mL DMF) dropped into 5 mL of toluene or BMA solvent. Schematic of the formation mechanism for Cs_4_PbBr_6_ and CsPbBr_3_ in the (**b**) prepolymer and (**c**) toluene anti-solvent systems.

Based on these results, the blue emission of prepolymer-0 originates from the quantum-confined amorphous CsPbBr_3_ nanoparticles, as described in previous reports [7,37]. To further investigate the formation mechanism of the Cs_4_PbBr_6_ nanoparticles in the prepolymer system, we focused on the difference in the solubility of PbBr_2_ between the prepolymer and toluene. The BMA monomer, which is a major component of the prepolymer system, is more polar than toluene (Figure S2), as evidenced by the dipole moments of their chemical structures of 2.173 and 0.516 D, respectively. This suggests stronger interactions between PbBr_2_ and the BMA monomer and higher solubility of PbBr_2_ in the monomer than those between PbBr_2_ and toluene, which exhibited weaker interactions and caused high precipitation, as shown in Figure 3a. As illustrated in Figure 3b,c, the difference in the solubility of PbBr_2_ in the prepolymer–DMF system was significantly smaller than that in toluene–DMF, whereas CsBr exhibited extremely low solubility in both the prepolymer and toluene. Therefore, when a precursor was dropped into the prepolymer anti-solvent, a relatively small amount of PbBr_2_ was precipitated from the prepolymer, whereas CsBr was mostly precipitated, resulting in the formation of Cs_4_PbBr_6_, which is a PbBr_2_ deficient phase, as the major component. The remaining CsBr and PbBr_2_ formed small amorphous CsPbBr_3_ nanoparticles, resulting in a blue emission. When the prepolymer temperature increased, the particle size of CsPbBr_3_ gradually increased to emit green light owing to the increased reaction rate. In contrast, as the solubility of PbBr_2_ and CsBr is extremely low in toluene, CsPbBr_3_ was mainly formed by maintaining the initial molar ratio of 1:1. Hence, Cs_4_PbBr_6_ can be easily generated in the prepolymer system containing BMA monomer.

**Figure 4:**
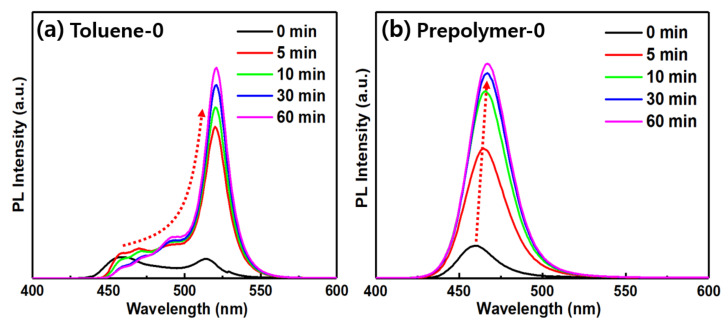
PL spectra as a function of reaction time after dropping in the precursor for the (**a**) toluene-0 and (**b**) prepolymer-0 samples.

**Figure 5:**
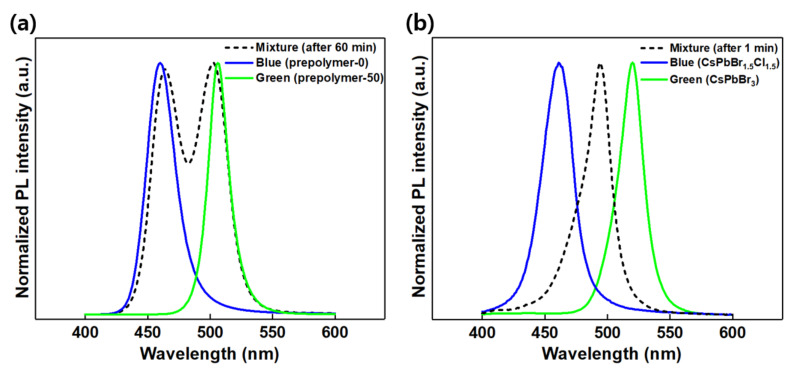
PL spectra for the color mixing tests of (**a**) prepolymer-0 (blue), prepolymer-50 (green), and a mixture of their solutions and (**b**) CsPbBr_1.5_Cl_1.5_ (blue) and CsPbBr_3_ (green) from the toluene anti-solvent and a mixture of their solutions.

The small CsPbBr_3_ NCs with blue PL emission are expected to be unstable owing to their large surface area [38]. This instability allows them to easily aggregate into large particles, thus redshifting their PL spectrum and giving rise to green emission, such as that for the toluene system shown in Figure 4a. However, our prepolymer system maintained its blue PL well in the solution over 1 h, as shown in Figure 4b, demonstrating the advantage of the prepolymer system. In addition, when the blue and green luminescent prepolymer-based solutions were mixed, two separate PL peaks persisted for more than 1 h, as shown in Figure 5a. This stability was induced by the fact that the blue and the green luminescent perovskite nanoparticles synthesized in this manner were both bromide compositions; thus, no ion exchange occurred between them. In contrast, upon mixing the two solutions of CsPbBr_1.5_Cl_1.5_ (blue) and CsPbBr_3_ (green) formed with toluene as the anti-solvent, their original peaks disappeared and merged into a new single peak at 490 nm owing to an anion exchange reaction between the two components (Figure 5b). This finding indicates the stability of the nanoparticles in maintaining their luminescent color even when the two solutions were mixed. Further, this demonstrates the higher potential of the prepolymer system in white-light or multicolor applications.

**Figure 6:**
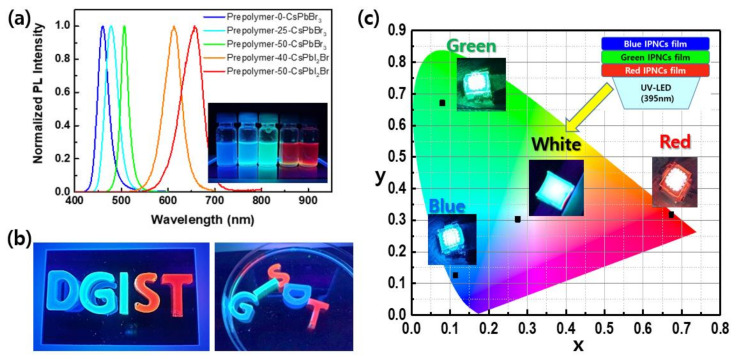
(**a**) Five different emission colors of the as-prepared IPeNC–prepolymer solutions and their PL spectra. (**b**) PL-emitting images of the prepared “DGIST” letters in ambient air (left) and in a water bath (right). (**c**) PL-emitting images of the color conversion films of red, green, blue, and white using a 395-nm UV light-emitting diode (LED), and their CIE color coordinates. The inset represents the configuration of the white LED device.

To express the three primary colors of RGB using the prepolymer system, a CsPbI_2_Br (1:1 molar ratio of CsBr:PbI_2_) precursor solution was injected into the prepolymer at temperatures of 40 and 50 °C to attain red emissions at 605 and 655 nm, respectively, as shown in Figure 6a. Finally, the IPeNC–prepolymer solution was coated on a substrate and exposed to UV light to fabricate water-stable PL-emitting composite films. This simplicity of the fabrication process is significantly advantageous for the preparation of the UV-curable prepolymer systems. As shown in Figure 6b, letters of different colors were produced through UV crosslinking. The letters stably emitted their PL color even in a water bath at an ambient temperature of 23 °C, confirming the passivation effect of the acryl-based prepolymer system after full crosslinking. The ability to freely form the final shape and emission color of the composite products is another notable advantage of the UV-curable prepolymer systems for their applications.

The IPeNCs are promising for display and lighting applications owing to their excellent optical properties [7,39,40]. RGB primary colors were well represented in the composite film state; thus, a wide color gamut of 98% of the National Television System Committee (NTSC) can be expressed, as shown in Figure 6c. A white-emitting film was fabricated by stacking the RGB color films on a 10 W UV LED. The correlated color temperature was 9700 K. These results show that this simple approach can be used to fabricate a color conversion layer for LCD backlight units and lighting systems.

## 4. Conclusions

We developed a facile method for synthesizing color-tunable all-bromide based perovskite NCs through a modified LARP method using UV-curable prepolymer as the anti-solvent. By controlling the prepolymer temperature, the PL color can be varied from blue to green without a Cl^−^ anion exchange process. After an in-depth study, we concluded that the stable blue emission of the sample obtained with a prepolymer temperature of 0 °C originates from the quantum-confined CsPbBr_3_ nanoparticles, which are nearly amorphous and approximately 4 nm in size. The selective solubility of the prepolymer to PbBr_2_ plays a key role in the formation of the all-bromide blue-emitting perovskite nanoparticles. Colorless, wide-bandgap Cs_4_PbBr_6_ nanoparticles with a PbBr_2_-deficient composition were initially generated owing to the selective solubility of the prepolymer. In addition, CsPbBr_3_ nanoparticles that had a radius smaller than the Bohr radius were formed, which emitted blue PL. Setting the temperature of the prepolymer higher increased both the size and crystallinity of CsPbBr_3_ nanoparticles, leading to green emission.

In terms of color stability, the synthesized all-bromide blue-emitting IPeNC solution remained stable, and two separate luminescence peaks persisted for over 1 h when mixed with the green-emitting IPeNC solution. By coating the IPeNC–prepolymer solutions and exposing them to UV light, we easily obtained a water-stable nanocomposite film with high emission quality and high potential for application as a color conversion layer in wide color gamut LCD backlight units and lighting lamp system.

## Data Availability

The data presented in this study are available on request from the corresponding author.

## Supplementary Materials

The following are available online at https://www.mdpi.com/article/10.3390/polym14030381/s1, Figure S1: Schematic of the synthesis of IPeNCs through the modified LARP method; Figure S2: Molecular structure and calculated dipole moments of chemicals used in the prepolymer preparation and toluene; Figure S3: Particle size distributions of the CsPbBr_3_ nanoparticles in (**a**) prepolymer-0, (**b**) prepolymer-25, (**c**) prepolymer-50, and (**d**) toluene-25 samples; Figure S4: Rietveld refinement of X-ray diffraction patterns of (**a**) prepolymer-0, (**b**) prepolymer-25, (**c**) prepolymer-50, and (**d**) toluene-25; Table S1: Compositional ratio of the samples calculated from the Rietveld refinements of the XRD patterns.
