# Supplementary material for: Wavelength-Tunable and Water-Stable Cesium–Lead-Based All-Bromide Nanocrystal–Polymer Composite Films Using Ultraviolet-Curable Prepolymer as an Anti-Solvent
Source: Polymers (Basel). 2022 Jan 19;14(3):381. doi: 10.3390/polym14030381 (PMC8840061; doi:10.3390/polym14030381)
Supplement: Supplementary file 1 [file polymers-14-00381-s001.zip › polymers-1501100-supplementary.pdf]

Supplementary Materials

Wavelength-Tunable and Water-Stable Cesium–Lead-Based All-Bromide Nanocrystal–Polymer Composite Films Using Ultraviolet-Curable Prepolymer as an Anti-Solvent

Wook Hyun Kim[1], Jungyoun Bae[1,2], Kang-Pil Kim[1], Sungho Woo[1,*]

[1] Division of Energy Technology, Daegu Gyeongbuk Institute of Science and Technology (DGIST), Daegu, 42988, Republic of Korea

[2] Department of Chemical Engineering, Kyungpook National University, Daegu, 41566, Republic of Korea

*Corresponding Author: shwoo@dgist.ac.kr

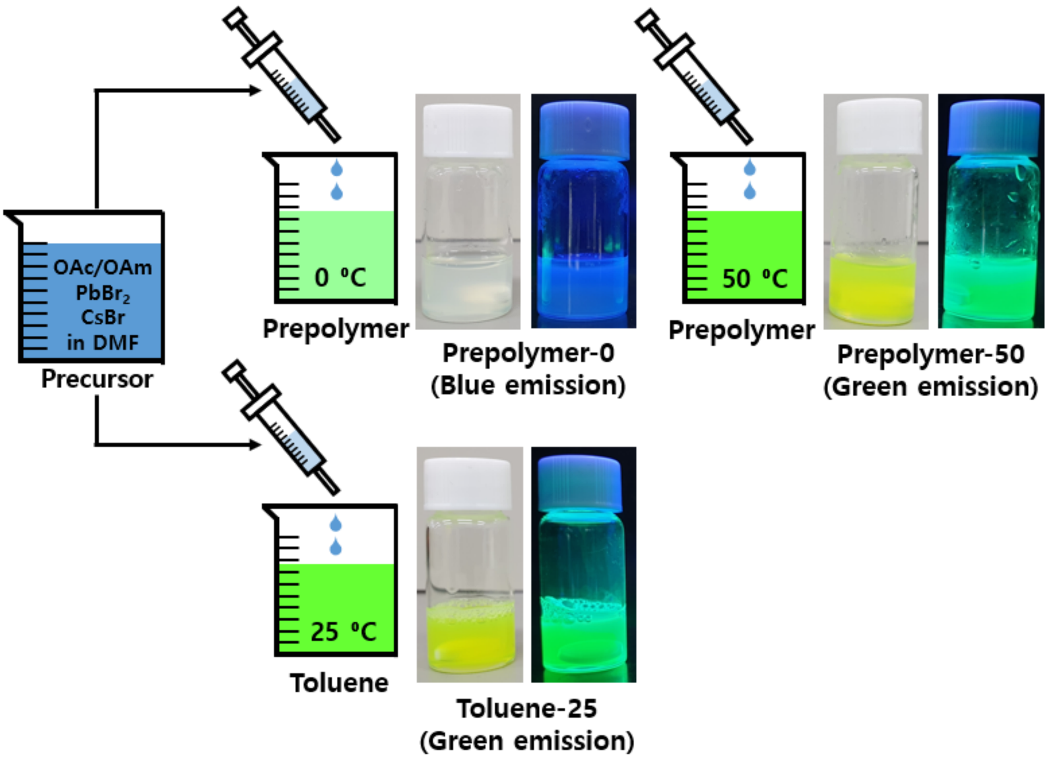

Figure S1. Schematic of the synthesis of IPeNCs through the modified LARP method.

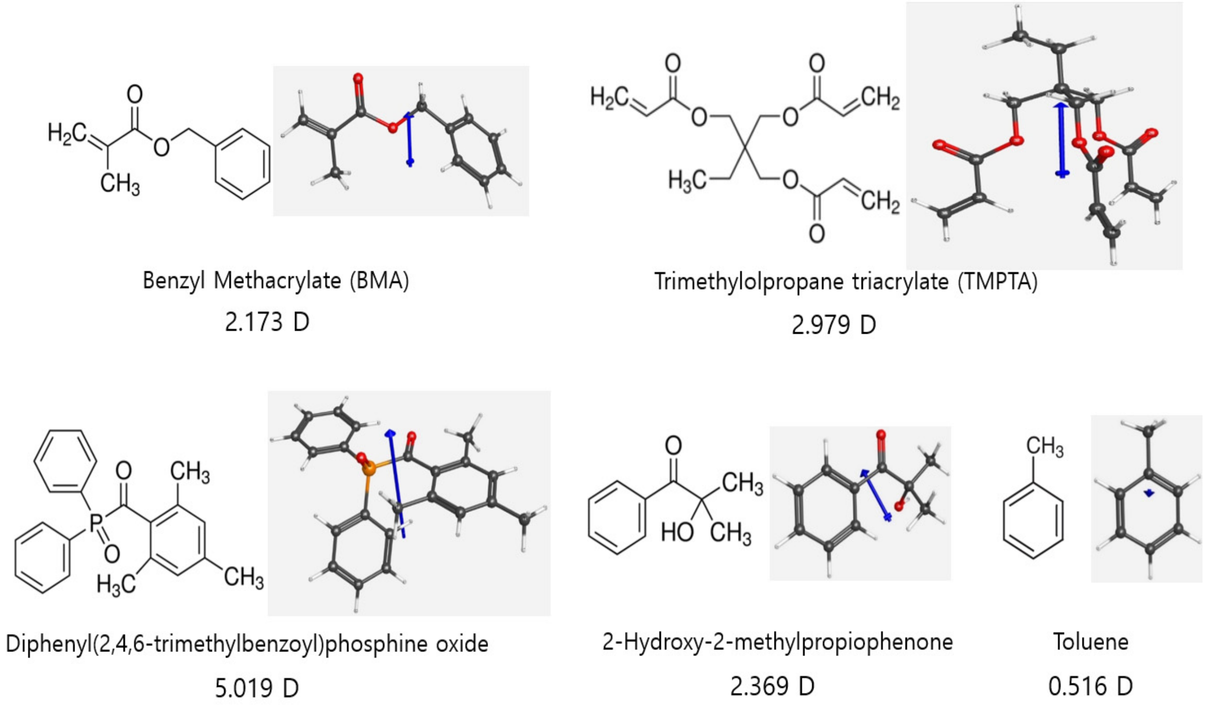

Figure S2. Molecular structure and calculated dipole moments of chemicals used in the prepolymer preparation and toluene.

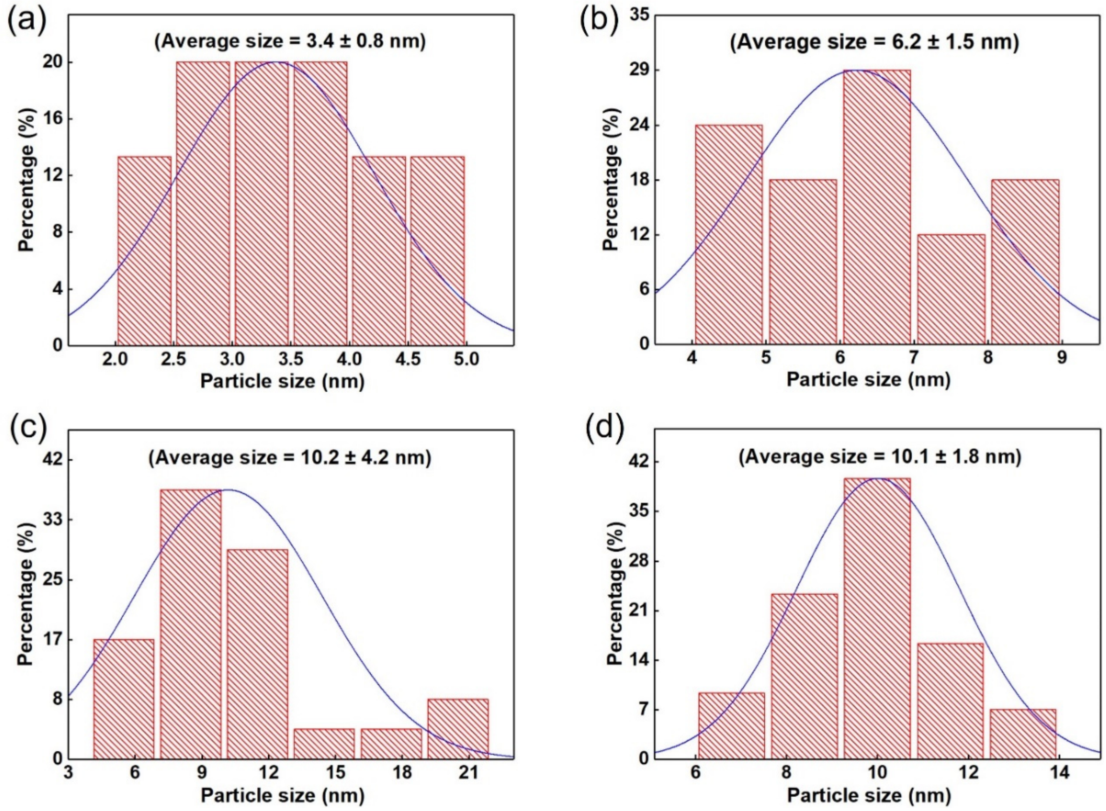

Figure S3. Particle size distributions of the $CsPbBr_3$ nanoparticles in the (**a**) prepolymer-0, (**b**) prepolymer-25, (**c**) prepolymer-50, and (**d**) toluene-25 samples.

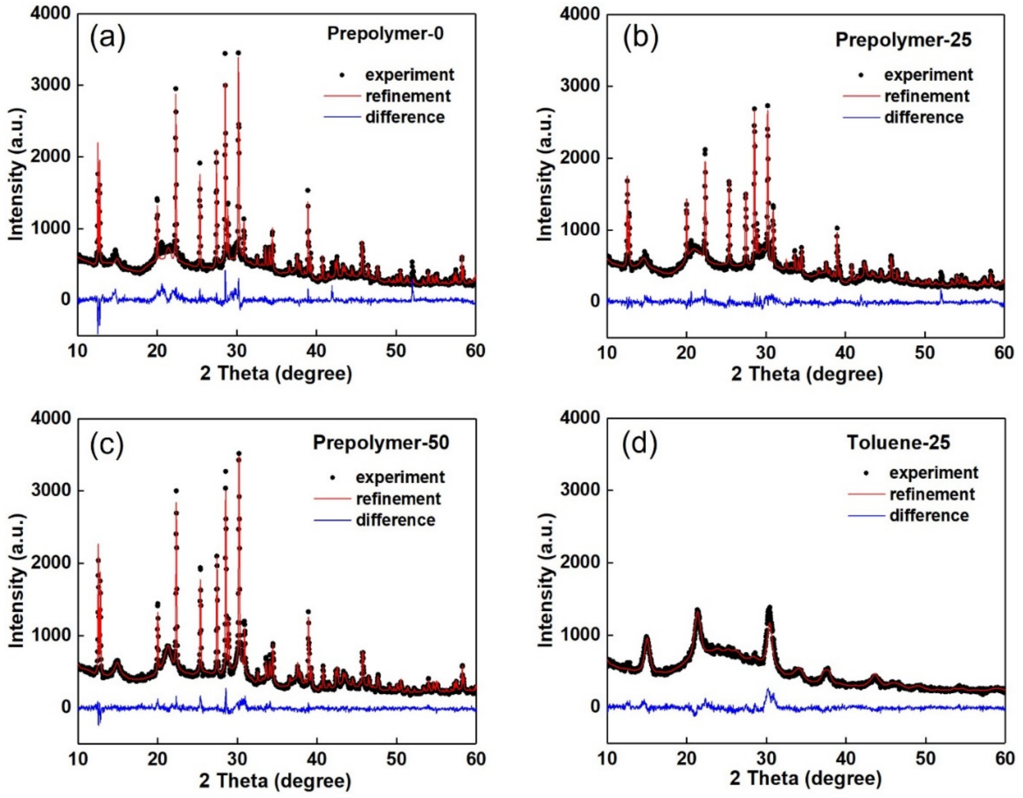

Figure S4. Rietveld refinement of XRD patterns of the (**a**) prepolymer-0, (**b**) prepolymer-25, (**c**) prepolymer-50, and (**d**) toluene-25 samples. Experimental (black dots), refined (red line), and difference (blue line) profiles obtained after the Rietveld refinement.

Table S1. Compositional ratio of the samples calculated from the Rietveld refinements of the XRD patterns. R_P, R_{WP}, and χ^2 denote the profile R-factor, weighted profile R-factor, and goodness of fit, respectively.

Samples	Calculated composition (%)		Refinement indices		
	Cs_4PbBr_6	$CsPbBr_3$	R_P (%)	R_{WP} (%)	χ^2
Prepolymer-0	87.3	12.7	5.9	8.4	3.27
Prepolymer-25	76.4	23.6	5.3	8.0	2.99
Prepolymer-50	73.5	26.5	4.7	6.0	1.65
Toluene-25	100	-	5.1	6.6	2.11
